# Ferroptosis-like cell death promotes and prolongs inflammation in *Drosophila*

**DOI:** 10.1038/s41556-024-01450-7

**Published:** 2024-06-25

**Authors:** Andrew J. Davidson, Rosalind Heron, Jyotirekha Das, Michael Overholtzer, Will Wood

**Affiliations:** 1 Wolfson Wohl Centre for Cancer Research, School of Cancer Sciences, College of Medical, Veterinary and Life Sciences, University of Glasgow, Glasgow, UK; 2 Institute for Regeneration and Repair, University of Edinburgh, Edinburgh, UK; 3 Cell Biology Program, Memorial Sloan Kettering Cancer Center, New York, NY USA; 4 Louis V. Gerstner, Jr. Graduate School of Biomedical Sciences, Memorial Sloan Kettering Cancer Center, New York, NY USA

**Keywords:** Cell death, Cellular imaging, Cell death and immune response, Inflammation

## Abstract

Ferroptosis is a distinct form of necrotic cell death caused by overwhelming lipid peroxidation, and emerging evidence indicates a major contribution to organ damage in multiple pathologies. However, ferroptosis has not yet been visualized in vivo due to a lack of specific probes, which has severely limited the study of how the immune system interacts with ferroptotic cells and how this process contributes to inflammation. Consequently, whether ferroptosis has a physiological role has remained a key outstanding question. Here we identify a distinct, ferroptotic-like, necrotic cell death occurring in vivo during wounding of the *Drosophila* embryo using live imaging. We further demonstrate that macrophages rapidly engage these necrotic cells within the embryo but struggle to engulf them, leading to prolonged, frustrated phagocytosis and frequent corpse disintegration. Conversely, suppression of the ferroptotic programme during wounding delays macrophage recruitment to the injury site, pointing to conflicting roles for ferroptosis during inflammation in vivo.

## Abstract (web summary)

Davidson et al. visualize ferroptosis-like cell death using three-colour live imaging in vivo and demonstrate its role in triggering macrophage recruitment but delaying resolution of inflammation during wounding in the *Drosophila* embryo.

## Main

Necrosis is a highly inflammatory type of cell death that occurs during tissue damage. In the past decade, ferroptosis has been identified as a distinct mode of necrosis, defined by iron-dependent lipid peroxidation^1–3^. Ferroptosis is an inherent vulnerability arising from the incorporation of volatile polyunsaturated lipids within the membranes of the cell^4–6^. As such, life has evolved a multi-faceted antioxidant defence system to prevent the accumulation of lipid peroxides, which, if left unchecked, inevitably triggers ferroptosis^7–10^. Despite increasing evidence of a role for ferroptosis in mammalian models of organ damage and cancer therapy, there has been no direct, in vivo visualization of ferroptosis^11–19^. Here, we show through three-colour live imaging that ferroptosis-like necrosis is a key component of acute injury, which is triggered during wounding of the *Drosophila* embryo. While we reveal a role for this ferroptotic programme in inflammation, the subsequent uptake of ferroptotic necrosis by macrophages is extremely poor. We propose that this distinct necrosis represents in vivo *Drosophila* ferroptosis. Ultimately, we propose that there are two sides to the role of ferroptosis during acute injury, which will require due consideration if ferroptosis is to be therapeutically targeted during organ damage and cancer treatment. On the one hand, it potently triggers inflammation, helping the tissue respond to damage. On the other hand, ferroptosis is inherently challenging to clear and vulnerable to counterproductive corpse disintegration, undoubtedly confounding the resolution of inflammation.

## Results

### Live, in vivo imaging of necrotic tissue damage

**Fig. 1:**
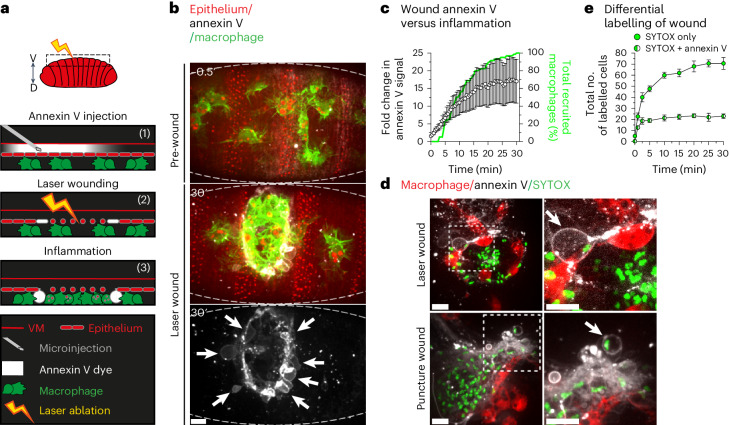
Three-colour, in vivo imaging of necrosis. **a**, Diagram highlighting laser-induced wounding in *Drosophila* embryo. The boxed region highlights region of *Drosophila* embryo (red) where laser ablation (yellow bolt) and imaging is performed. The ventral (V) and dorsal (D) axis is indicated. The protocol for three-colour imaging of laser-induced tissue damage is indicated in 1–3. (1) Far-red annexin V dye (white) is microinjected through the vitelline membrane (VM, red line) into intervitelline space apical to mcherry-labelled embryonic epithelium (red ovals). The GFP-labelled macrophages (green) immediately below the epithelium are not exposed to annexin V or damage caused by microinjection. (2) Laser ablation (yellow bolt) of the epithelium induces tissue damage, together with annexin V labelling (white) of cells at the wound edge. (3) During inflammation, the macrophages are recruited to clear cellular debris at the wound, which is ringed by extruded, annexin V-labelled epithelial cells. **b**, Three-colour imaging of laser-induced wounding. Top: the ventral epithelium of the embryo (outlined) is injected with far-red annexin V (white), pre-laser ablation. Macrophages (lifeact-GFP, green) are evenly dispersed underneath ventral epithelium (mcherry-moesin, red). Middle: the same embryo 30 min post-laser ablation. Laser ablation induces inflammatory recruitment of macrophages to the wound and annexin V labelling of cells around the wound edge. Bottom: same as above but only showing annexin V labelling of extruded cells around the wound edge (arrows). Scale bar, 10 µm. **c**, The wound edge is rapidly labelled with annexin V (white circles, fold change plotted on left hand *Y* axis; *N* = 6 wounded embryos), correlating with macrophage recruitment (green line, per cent total plotted on right hand *Y* axis; *N* = 6 wounded embryos). The error bars are the mean ± standard error of the mean (s.e.m.). **d**, Distinct pattern of necrosis revealed within wounds. Wounded (laser ablation (18 min post-wounding) (top), needle puncture (bottom)) ventral epithelium of embryos co-injected with far-red annexin V (white) and SYTOX (necrotic dye, green). The macrophages are labelled with mcherry (red). The dashed boxes magnified to highlight double-labelled cells (arrows) at the wound edges. Scale bar, 10 µm. **e**, Differential labelling of size-matched wounds (SYTOX only (solid green circles), SYTOX and annexin V (green and white circles)) in the aftermath of laser ablation (*N* = 3 wounded embryos, error bars are the mean ± s.e.m.). Source data

**Extended Data Fig. 1:**
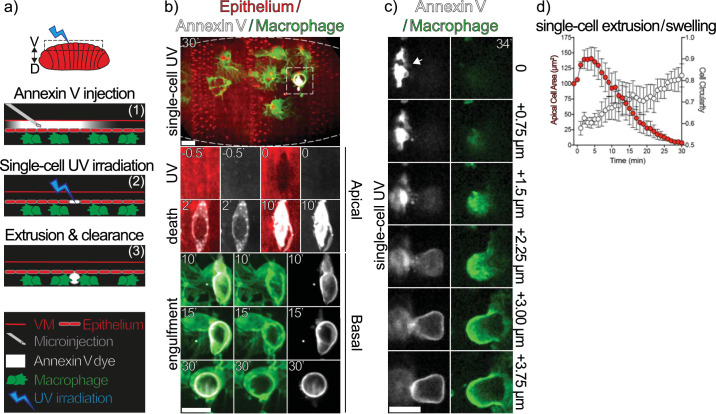
3-colour imaging of UV-induced, single-cell death. **1a**) Diagram highlighting UV-induced, single-cell death in *Drosophila* embryo. TOP: Boxed region highlights region of *Drosophila* embryo (red) where single-cell UV irradiation (blue bolt) and imaging occurs. Ventral (V) and Dorsal (D) axis is indicated. PANELS 1-3: protocol for 3-colour imaging of UV-induced, single-cell death. (1) Far-red Annexin V dye (white) is microinjected through vitelline membrane (VM, red line) into intervitelline space immediately apical to mcherry-labelled embryonic epithelium (red ovals). The GFP-labelled macrophages (green) immediately below the epithelium are not exposed to Annexin V or damage. (2) Targeted UV-irradiation (blue bolt) of single epithelial cell induces cell death, triggering Annexin V labelling. (3) The irradiated cell is extruded basally from epithelium and engulfed by recruited macrophages. **1b**) 3-colour imaging of UV-induced, single-cell death. TOP: Overview of ventral epithelium of injected embryo (outlined) 30 mins after single-cell UV irradiation (dashed box). The surrounding epithelium (mcherry-moesin, red) extrudes the far-red Annexin V labelled irradiated cell (white), which is engulfed by a macrophage (lifeact-GFP, green). MIDDLE PANELS: Key time points (mins) before and after UV-irradiation of single epithelial cell. The irradiated cell is specifically labelled with Annexin V within 2 min and extrusion occurs by 10 min. LOWER PANELS: The same cell as above, viewed basally from 10–30 min post-UV. Immediately upon extrusion, macrophages are recruited to engulf irradiated cell corpse. Scale bars =10 µm. **1c**) UV-irradiated cells remain tethered to epithelium after extrusion, resulting in residual, uncleared debris after engulfment. Unprojected, individual z-slices from the same Annexin V labelled (white), engulfed corpse from Extended Data Fig. 1b are shown (34 mins post-irradiation), from the apical surface of epithelium to the underlying macrophage (GFP-moesin, green) in 0.75 µm increments. Post-engulfment, Annexin V positive debris originating from the epithelial tether (z = 0) is left behind as a ‘scar’ (arrow). The rest of the corpse is fully enclosed within the macrophage (z = 0.75–2.25 µm), which at this time point is moving away from the site of engulfment. Scale bar =10 µm. **1d**) Cell extrusion and swelling during UV-induced, single-cell death. Immediately post-UV, the apical area of the epithelium expands, before rapidly decreasing during extrusion (red circles, plotted on left hand Y-axis, n = 5 cells). Conversely, the circularity of the Annexin V labelled cell increases indicative of cellular swelling (white circles, plotted on right hand Y-axis, n = 8 cells). Error bars =mean ± SEM. Source data

**Extended Data Fig. 2:**
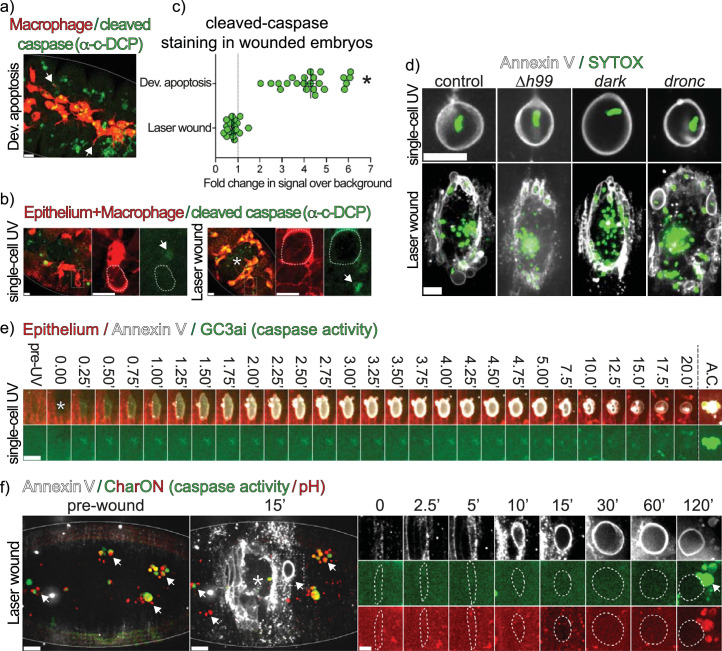
Annexin V-labelled death triggered by UV/wounding is nonapoptotic. **2a–c**) The cleavage-mediated activation of the *Drosophila* caspase, DCP-1, is a defining feature of fly apoptosis and is detectable by immunohistochemistry. **a**) Cleaved-caspase staining of developmental apoptosis. Ventrally dispersing macrophages (mcherry, red) clear developmental apoptosis (cleaved-caspase (anti-c-DCP-1), green) occurring within the CNS (embryonic stage 13). Arrows highlight examples of macrophage mediated clearance via filopodial phagocytosis. Scale bar =10 µm. **b**) There is no caspase activation during (LEFT) UV-induced, single-cell death or (RIGHT) laser-induced wounding. In contrast to developmental apoptosis within CNS of embryo (arrows), no cleaved-caspase (anti-c-DCP-1, green) is detected during UV-induced, single-cell death within epithelium (mcherry-moesin, red) or around edge of wound. Cell corpses (dashed outlines) identified through enveloping macrophages (mcherry, red). Dashed box in embryo overviews enlarged in adjacent panels. Scale bars =10 µm. **c**) Fold-change in cleaved caspase signal (anti-c-DCP-1) over background (=1, dashed line) in enveloped corpses surrounding wounds compared to developmental apoptosis found within the same embryos. Developmental apoptosis exhibits significantly higher signal (asterisk, unpaired t test, p < 0.0001) compared to corpses surrounding the wound. Signal in developmental apoptosis significantly greater than background (one sample t and Wilcoxon test, p < 0.0001), while signal in corpses surrounding wound is significantly less than background (one sample t and Wilcoxon test, p < 0.01). Error bars =mean ± SEM, n ≥ 16 corpses per group, N = 7 wounded embryos. **2d**) UV-induced, single-cell death and wounds in mutants for key apoptotic regulators. SYTOX (green) and Annexin V (white) labelling of UV-induced, single-cell death (TOP) and laser wounds (BOTTOM) is unperturbed in *Drosophila* mutants of *∆h99* (lack Reaper family genes), *dark* (APAF1 homolog) and *dronc* (apical caspase). Cells/wounds shown 20 min post-UV, scale bars =10 µm. **2e**) No caspase activation is detected by the GC3ai biosensor during UV-induced, single-cell death. UV-irradiation (asterisk) of individual cell of epithelium (mcherry-moesin, red), triggers rapid labelling with Annexin V (white), but no detectable caspase activation as measured by GC3ai biosensor. As a comparison, a *bona fide* apoptotic corpse (A. C.) from within the embryonic CNS is strongly labelled by both Annexin V and GC3ai. Time =minutes, scale bar =10 µm. **1f**) No caspase activation is detected by the CharON biosensor during laser-induced wounding. CharON consists of a caspase-activated GFP (GC3ai-based) and an acid-activated RFP. LEFT: Overview of CharON (green/red) expressing embryo (outlined) injected with far-red Annexin V (white), shown pre-wound. At developmental stage (embryonic stage 15) shown, all of the ventral most apoptotic corpses are internalised with unlabelled macrophages (arrowed clusters) and are transiting through the phagolysosomal system (that is increasingly red). MIDDLE: Same embryo shown 15 minutes post-wounding (asterisk). Laser-ablation induces strong Annexin V labelling of wound edge in the absence of caspase activation (that is green signal). Boxed, Annexin V positive cell at edge of the wound shown over indicated time range in adjacent inserts (RIGHT). No caspase activity or acidification was detected by CharON within the cell (outlined) up to 2 hours post-wounding. Arrow highlights engulfed apoptotic corpses in unlabelled macrophage recruited as part of inflammation. Time = minutes, scale bars 10/5 µm. Source data

**Extended Data Fig. 3:**
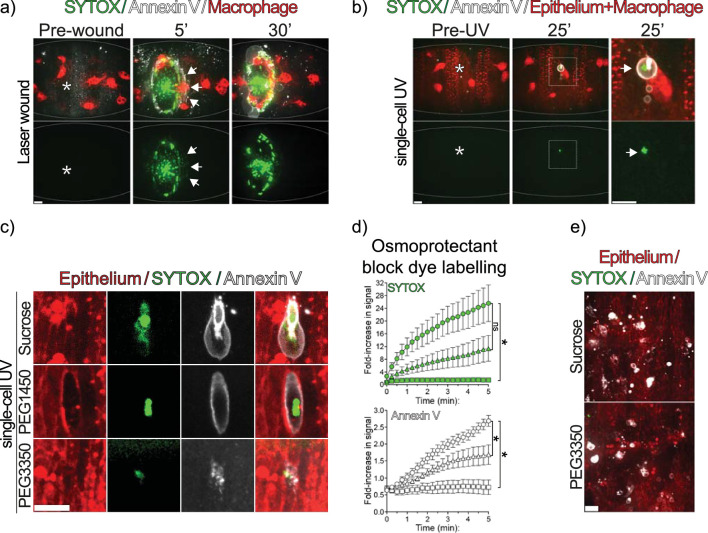
SYTOX uptake during Annexin V positive necrosis. **3a**) SYTOX labels Annexin V positive cell death surrounding wound. Embryos were co-injected with SYTOX (green) and far-red Annexin V (white) and wounded by laser-ablation (asterisk), resulting in necrotic tissue damage and inflammatory macrophage (mcherry, red) recruitment. Pre-extrusion (5 min after laser-ablation), cells surrounding the wound co-label for both Annexin V and SYTOX (arrows). Subsequent extrusion results in these cells ‘ballooning’ away from edge of wound in X, Y and Z planes. Note: There is no detectable SYTOX labelling in embryo pre-wounding. Time = minutes, scale bar = 10 µm. **3b**) UV-induced, single-cell death is necrotic. LEFT and MIDDLE: Overview of the ventral face of embryo (epithelium and macrophages labelled with mcherry, red) co-injected with far-red Annexin V (white) and SYTOX (necrotic dye, green), shown pre-UV and 25 min after irradiation. RIGHT: Higher magnification of boxed region in above. Strong labelling of irradiated cell with both Annexin V and SYTOX (arrow), accompanied by macrophage recruitment. Note: There is no detectable SYTOX labelling in embryo pre-UV. Time = minutes, scale bars = 10 µm. **3c–e**) Osmoprotectants block swelling and both SYTOX and Annexin V labelling during UV-induced, single-cell death. (**c**) In contrast to injected sucrose (control), high molecular weight PEG3350 strongly suppresses SYTOX (green) and Annexin V (white) labelling during UV-induced necrosis of individual cell of ventral epithelium (mcherry-moesin, red). Cell swelling and extrusion also inhibited by high molecular weight PEG. PEG1450 with intermediate molecular weight causes partial inhibition. Brightness/contrast kept constant across all SYTOX and Annexin V panels. As such, the SYTOX signal is saturating in control and PEG1450 images in order to detect SYTOX in PEG3550 image. Cells shown at 15 minutes post-UV, scale bars =10 µm. (**d**) TOP: Fold-change in SYTOX uptake in immediate aftermath of single-cell necrosis in embryos injected with sucrose (control, circles), PEG1450 (triangles) or PEG3550 (squares). High (but not intermediate) molecular weight PEG significantly suppresses SYTOX uptake (asterisk, tested at 5 minute time point, one-way ANOVA with Dunnett’s comparisons test, padj < 0.05, ns = nonsignificant, p = 0.0526). BOTTOM: Fold-change in Annexin V labelling in immediate aftermath of single-cell necrosis in embryos injected with sucrose (control, circles), PEG1450 (triangles) or PEG3550 (squares). Both intermediate and high molecular weight PEGs suppress Annexin V labelling (asterisks, tested at 5 min time point, one-way ANOVA with Dunnett’s comparisons test, padj < 0.05). Error bars = mean ± SEM. N ≥ 5 embryos/injection regime. (**e**) Osmoprotectants do not inhibit Annexin V labelling of apoptosis. Microinjection of SYTOX (green), far-red Annexin V (white) and either sucrose or PEG3550 into embryonic CNS (basal to labelled epithelium, mcherry-moesin, red) results in strong Annexin V (but not SYTOX) labelling of developmental apoptosis (embryonic stage 13). Scale bar = 10 µm. Source data

The *Drosophila* embryo provides a powerful model system to live image fundamental cell processes within the complex setting of a living animal. Recent studies have used this system to shed new light on the intricacies of developmental apoptosis and its clearance during *Drosophila* embryogenesis, but little is known about the dynamics of non-developmental cell death and its engulfment in vivo^20^. We have previously shown that laser-induced wounding of *Drosophila* embryos leads to extensive tissue damage that triggers a rapid inflammatory response from macrophages^20–22^. To investigate the molecular nature of this tissue damage in more detail, we microinjected far-red annexin V into *Drosophila* embryos, and through three-colour live imaging, we revealed a distinct ring of annexin V-labelled cell death at the edge of the wound, the appearance of which correlated with inflammation (Fig. 1a–c and Supplementary Video 1). Morphologically, this wound-induced cell death appeared identical to the ultraviolet (UV)-induced, annexin V-positive single-cell death, previously considered apoptotic (Extended Data Fig. 1a–d and Supplementary Video 2)^22–24^. However, wounds generated by laser ablation in the embryo are entirely necrotic^20,22^. Therefore, we hypothesized that this cell death was, in fact, a distinct form of necrosis. We excluded any mechanistic role for the apoptotic programme in this process (Extended Data Fig. 2a–f), and the lack of *Drosophila* homologues to MLKL or the gasdermins discounts the possibility that this necrosis might be necroptotic or pyroptotic. Furthermore, while it has been suggested that *Drosophila* have a ‘necroptosis-like’ cell death, this is dependent on the apical caspase, Dronc, which is not required for the death identified here (Extended Data Fig. 2d)^25^. To first confirm that this cell death was necrotic, we co-injected far-red annexin V with SYTOX, a membrane impermeable dye taken up during necrotic lysis. Strikingly, when these dyes were used to label wounded tissue, a distinct pattern of necrosis was revealed. In laser-induced wounds, a SYTOX-high, annexin V-low necrotic core was ringed by distinct, double-labelled necrotic cells (Fig. 1d,e, Extended Data Fig. 3a and Supplementary Video 3) with the latter accounting for ~30% of the necrosis in laser wounds (Fig. 1e). Importantly, this double-labelled necrosis was also triggered by laser-free, puncture wounding using a needle (Fig. 1d). UV-induced, single-cell death was also rapidly and invariably co-labelled by both SYTOX and annexin V (Extended Data Fig. 3b–d and Supplementary Video 4). We concluded that we had identified a distinct type of necrosis occurring during acute injury, distinguishable in vivo through annexin V labelling.

Annexin V labelling is conventionally associated with phosphatidylserine externalization during apoptosis^26^. However, it has been long known (although often over looked) that annexin V also labels necrosis^27^. To examine if necrotic cell swelling was involved in the annexin V labelling we observed, we co-injected SYTOX and annexin V along with osmoprotectants into embryos and performed UV-induced, single-cell necrosis^28^. Osmoprotectants blocked necrotic swelling and also inhibited SYTOX and annexin V labelling (Extended Data Fig. 3c,d), suggesting that necrotic cell swelling alters the plasma membrane and leads to annexin V labelling. Conversely, osmoprotectants did not block annexin V labelling of developmental apoptosis (Extended Data Fig. 3e). We concluded that annexin V labelling indicated a distinct form of necrosis triggered during tissue damage.

### Ferroptotic-like necrosis is triggered during acute injury

**Fig. 2:**
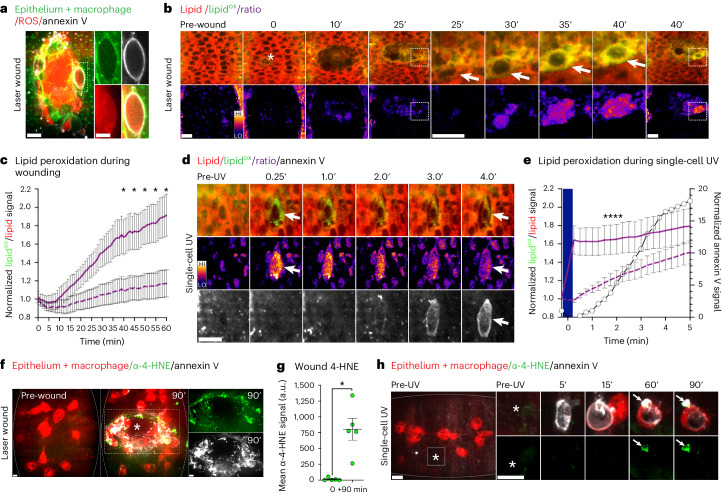
In vivo lipid peroxidation during acute injury. **a**, Three-colour, in vivo imaging of ROS within wounds. Laser-wounded embryo co-injected with far-red annexin V (white) and Amplex Red (ROS, red). Embryonic epithelium and macrophages (GFP-moesin) are designated in green. The dashed box is magnified in the accompanying inserts. Scale bars, 10 and 5 µm. **b**,**c**, Live imaging of lipid peroxidation within wounds. Timelapse imaging of laser-wounded (asterisk) ventral epithelium of embryo injected with ratiometric BODIPY^581/591^ C11 dye (**b**). Top: red–green shift of BODIPY^581/591^ C11 indicates lipid peroxidation. The dashed boxes (magnified in intervening panels) highlight the cell at the wound edge (arrow), which exhibits intense lipid peroxidation 25–30 min after initial wounding. Bottom: ratiometric (lipid^ox^/lipid (green–red)) heat maps corresponding to above images. Time is indicated in minutes. Scale bars, 10 µm. Lipid peroxidation (lipid^ox^/lipid) in cells ringing edge of wound (solid line) versus undamaged cells in surrounding tissue (dashed line) (**c**). The asterisks indicate the statistically significant difference at indicated timepoints (compared at 5 min intervals, one-way ANOVA with Sidak’s multiple comparisons test; *P*_adj_ <0.05, *n* = 6 cells per region, *N* = 3 wounded embryos). The error bars are the mean ± standard error of the mean (s.e.m.). **d**,**e**, Lipid peroxidation precedes annexin V labelling during UV-induced, single-cell necrosis. Three-colour imaging of UV-irradiated epithelial cell (arrows) in embryo co-injected with ratiometric BODIPY^581/591^ (green–red) and far-red annexin V (white) (**d**). Top: red–green shift of BODIPY^581/591^ C11 dye occurs immediately post-UV. Middle: ratiometric (lipid^ox^/lipid (green–red)) heat maps corresponding to above images. Bottom: annexin V labelling of irradiated cell occurs minutes post-UV. Scale bar, 10 µm. Lipid peroxidation (lipid^ox^/lipid) in irradiated cells (solid purple line) versus non-irradiated cells in surrounding tissue (dashed purple line), alongside the annexin V labelling of the irradiated cells (white circles, plotted on right hand *Y* axis) (**e**). The blue rectangle delineates UV irradiation. The asterisks indicate statistical significance (compared at 0.25, 0.5, 0.75, 1, 1.5, 2, 3, 4 and 5 min, one-way ANOVA with Sidak’s multiple comparison test; *P*_adj_ <0.05, *n* = 9 cells per region, *N* = 9 embryos). The error bars are the mean ± s.e.m. **f**, Three-colour imaging of 4-HNE accumulation after laser wounding of ventral epithelium (mcherry-moesin,) of embryo injected with far-red annexin V (white) and anti-4-HNE (fluorescent primary antibody, green). The macrophages are labelled with mcherry (red). Left: no 4-HNE is detected in pre-wounded embryo. Middle: 4-HNE detected around annexin V-labelled, wound (asterisk) edge 90 min post-wounding. Right: single-colour images of boxed region in above. Scale bars, 5 µm. **g**, Levels of 4-HNE at wound edge are significantly (asterisk) increased 90 min after laser wounding (Mann–Whitney test, *P* = 0.0079, *N* = 5 wounded embryos). a.u., arbitrary units. The error bars are the mean ± s.e.m. **h**, Three-colour imaging of a UV-irradiated cell in the ventral epithelium (mcherry-moesin, red) of an embryo co-injected with far-red annexin V (white) and anti-4-HNE (fluorescent primary antibody, green). The macrophages are labelled with mcherry (red). Left: embryo overview, pre-irradiation. The boxed region highlights the area of irradiation (asterisk), which is magnified in subsequent panels. Right: annexin V-labelled irradiated cell is rapidly enveloped and internalized by the macrophage, limiting labelling. 4-HNE is detected (arrows) 60–90 min post-UV at unengulfed annexin V-positive debris. Time is indicated in minutes. Scale bars, 10 µm. Source data

**Extended Data Fig. 4:**
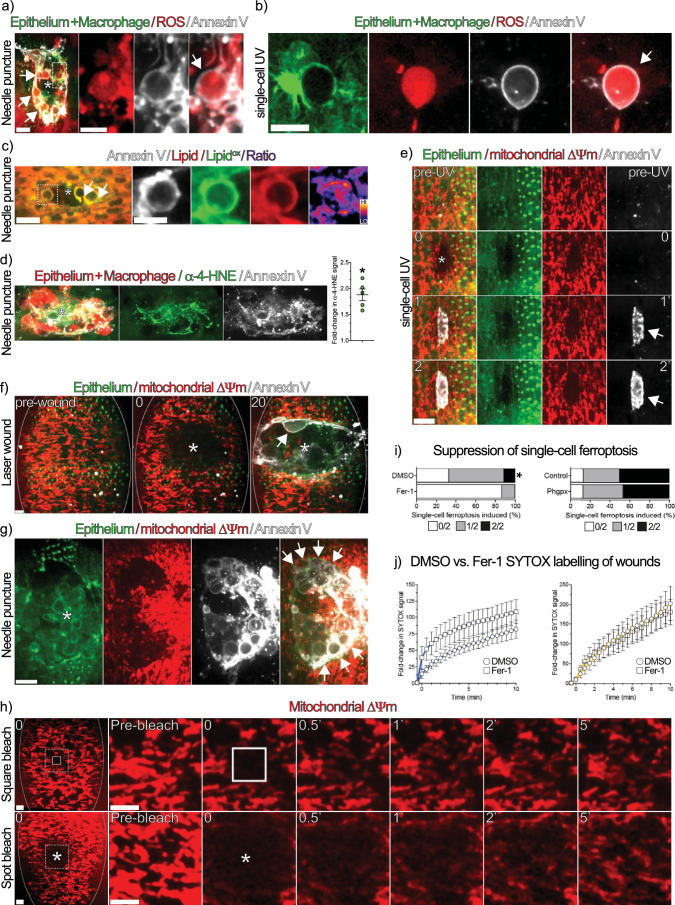
Hallmarks of ferroptosis during acute injury. **4a**) 3-colour, *in vivo* imaging of ROS generation during laser-free wounding. Needle punctured (asterisk) embryo co-injected with far-red Annexin V (white) and Amplex Red (ROS, red). Embryonic epithelium and macrophages labelled with GFP-moesin (green). Dashed box highlighting Annexin V positive necrosis at wound edge is magnified in accompanying inserts. Scale bars =10/5 µm. **4b**) 3-colour, *in vivo* imaging of intracellular ROS generation during UV-induced, single cell necrosis. Embryo co-injected with far-red Annexin V (white) and Amplex Red (ROS, red) followed by UV-irradiation of individual epithelial cell. UV triggers Annexin V positive necrosis and intracellular ROS generation (arrow). Embryonic epithelium and macrophages labelled with GFP-moesin (green). Time =10 minutes post-UV. Scale bars =10 µm. **4c**) Lipid peroxidation during laser-free wounding. Needle punctured (asterisk) ventral epithelium of embryo co-injected with ratiometric BODIPY^581/591^ C11 dye (green/red) and far-red Annexin V (white). LEFT: Overview of wound, wherein the red-green shift of BODIPY^581/591^ C11 indicates lipid peroxidation (lipid^ox^). Arrows highlight cells at wound edge exhibiting high lipid peroxidation. Dashed box (magnified in intervening panels) highlights two cells at wound edge (arrow) exhibiting intense lipid. Third such corpse (dashed box) magnified in adjacent panels. MIDDLE PANELS: Lipid peroxidation of Annexin V positive corpse at wound edge. RIGHT: Ratiometric (Lipid^ox^/Lipid (green/red)) heat map confirms lipid peroxidation within cell. Scale bars = 10/5 µm. **4d**) LEFT: 3-colour imaging of 4-HNE accumulation after needle puncture of ventral epithelium (mcherry-moesin,) of embryo injected with far-red Annexin V (white) and anti-4-HNE (fluorescent primary antibody, green). Macrophages labelled with mcherry (red). RIGHT: 4-HNE levels at needle puncture wounds (30 min post-wounding) are significantly higher than embryo-wide signal (asterisk, One sample t and Wilcoxon test, p < 0.005). N = 5 wounded embryos. **4e**) 3-colour, *in vivo* imaging of mitochondrial depolarisation during UV-induced, single cell necrosis. Embryo co-injected with far-red Annexin V (white) and TMRE dye (mitochondrial polarisation (∆Ψm), red), followed by UV-irradiation (asterisk) of individual cell of epithelium (GFP-moesin, green). UV triggers immediate mitochondrial depolarisation, preceding Annexin V labelling (arrow). Time = minutes, scale bar 10 µm. **4f**) 3-colour, *in vivo* imaging of mitochondrial depolarisation in laser-induced wound. Embryo co-injected with far-red Annexin V (white) and TMRE dye (mitochondrial polarisation (∆Ψm), red) and shown pre-wound (LEFT) or 0 (MIDDLE) or 20 mins (RIGHT) after laser-ablation (asterisk) of ventral epithelium (GFP-moesin, green). Mitochondrial depolarisation evident throughout wound, including within highlighted Annexin V positive corpse (arrow). Scale bar =5 µm. **4g**) 3-colour, *in vivo* imaging of mitochondrial depolarisation during laser-free wounding. Embryo co-injected with far-red Annexin V (white) and TMRE dye (mitochondrial polarisation (∆Ψm), red), followed by needle puncture (asterisk). Of ventral epithelium (GFP-moesin, green). Mitochondrial depolarisation evident throughout wound, especially in Annexin V positive corpses (arrows). Scale bar =10 µm. **4h**) Loss of TMRE signal during UV-induced, single-cell death is not due to photobleaching alone. Embryos were injected with TMRE dye (mitochondrial polarisation (∆Ψm), red) and a 561 nm laser was used to photobleach either a square (solid white square, TOP ROW) or spot (asterisk, BOTTOM ROW) region on the ventral side of the embryo. LEFT: Overview of photobleached (time = 0) embryos (outlined in white). Dashed boxes magnified in subsequent panels. RIGHT: Time series of highlighted region, pre- and post-photobleaching. Regardless of bleach protocol, fluorescence recovery was rapid, implying sustained TMRE loss is due to mitochondrial depolarisation rather than photobleaching. Note: Photobleaching of the square region was restricted to the minimum time required for loss of TMRE signal. In contrast, the spot photobleaching recapitulated the longer irradiation required to trigger single-cell ferroptotic necrosis using UV (hence the wide area photobleached). Time =minutes, scale bars =5 µm. **4i**) Injection of Fer-1 (LEFT), but not overexpression of GPX4 homolog, Phgpx (RIGHT), is sufficient to significantly supress (asterisk, Mann-Whitney test, p < 0.01) UV-induced, single-cell ferroptotic necrosis. Two cells irradiated per embryo, N ≥ 9 embryos per injection regime. **4j**) Fer-1 does not block necrosis *per se* during laser-induced wounding. Fold-change in SYTOX signal within wound area (LEFT, N = 8 wounded embryos/drug) or within individual cells at wound edge (RIGHT, n = 24 cells, N = 8 wounded embryos/drug) in embryos injected with either DMSO (circles) or Fer-1 (squares). Error bars = mean ± SEM. No significant differences detected (tested at 2.5 min intervals, Krustal-Wallis tests, with Dunn’s multiple comparison tests, p > 0.05 in all cases). Note: same wounded embryos and cells analysed in Fig. 3d–f. Source data

We next investigated whether the distinct, double-labelled necrosis represented an in vivo form of an established mode of regulated necrosis. During wounding of the *Drosophila* embryo and other organisms, high levels of reactive oxygen species (ROS) are generated^29–31^. Using our three-colour imaging protocol, we observed ROS within the annexin V-positive necrotic cells ringing the edge of both laser-induced and needle puncture wounds (Fig. 2a and Extended Data Fig. 4a). We then confirmed the same intracellular generation of ROS following UV-induced, single-cell necrosis (Extended Data Fig. 4b). ROS is toxic to cells in many ways and is implicated in diverse types of cell death^3^. One distinct example is its role in fuelling the Fenton chemistry that drives lipid peroxidation, the causative agent of ferroptosis. To live image lipid peroxidation during acute injury, we microinjected the ratiometric BODIPY^581/591^ C11 dye into embryos before wounding^32^. This revealed robust lipid peroxidation within individual necrotic cells at the wound (Fig. 2b). Laser ablation induced lipid peroxidation throughout the wound, which was particularly pronounced around the wound edge (Fig. 2b,c and Supplementary Video 5). Lipid peroxidation was also evident during UV-induced, single-cell necrosis and, crucially, during laser-free, puncture wounding (Fig. 2d,e and Extended Data Fig. 4c). Both lipid peroxidation and cell death were often but not always triggered immediately upon wounding. Instead, a spike in lipid peroxidation and the associated cell death often occurred tens of minutes after the initial injury, suggesting they were a distinct event from the primary injury (Fig. 2b). Additionally, lipid peroxidation generates lipid–protein adducts, which can be detected by staining for 4-hydroxynonenal (4-HNE). We made use of a fluorescent anti-4-HNE primary antibody to confirm laser wounding triggered lipid peroxidation in live tissue (Fig. 2f,g). Strong 4-HNE labelling accumulated around the edge of wounds, co-localizing with annexin V-positive necrosis. 4-HNE was also detected in wounds made by needle puncture, as well as during UV-induced, single-cell necrosis (Fig. 2h and Extended Data Fig. 4d).

**Fig. 3:**
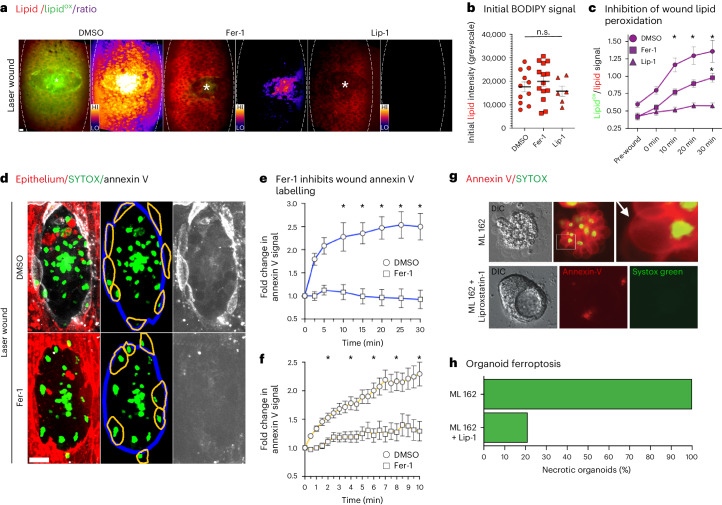
There is a ferroptotic component to acute injury. **a**, Inhibition of wound-induced lipid peroxidation. **a**, Ventral epithelium of laser-wounded (asterisks) embryos (outlined) co-injected with ratiometric BODIPY^581/591^ C11 dye (red–green shift indicates lipid peroxidation) and DMSO, ferrostatin-1 (Fer-1) or liproxstatin-1 (Lip-1). All embryos are 20 min post-wound. The images are paired with the corresponding heat map. Scale bar, 10 µm. **b**, No significant difference (n.s.) detected in initial (pre-wound) BODIPY^581/591^ C11 red signal in embryos co-injected with DMSO, Fer-1 or Lip-1 (one-way ANOVA with Tukey’s multiple comparison test, p_adj_ > 0.05, N ≥ 6 wounded embryos). The error bars are the mean ± standard error of the mean (s.e.m.). **c**, Inhibition of lipid peroxidation during wounding. BODIPY^581/591^ C11 lipid^ox^/lipid (green–red) signal pre- and post-wounding in embryos co-injected with DMSO (circles), Fer-1 (squares) or Lip-1 (triangles). The asterisks indicate statistically significant difference compared to pre-wound DMSO at indicated timepoints (Kruskal–Wallis tests with Dunn’s multiple comparisons tests; *P*_adj_ <0.05, *N* ≥ 6 wounded embryos). The error bars are the mean ± s.e.m. **d**, The effect of inhibiting lipid peroxidation on wound-induced necrosis. Top: laser-wounded ventral epithelium (mcherry-moesin, red) of embryos co-injected with far-red Annexin V (white), SYTOX (green) and DMSO (top) or Fer-1 (bottom). Middle: SYTOX only signal and outlines of wound (blue) and wound-edge necrotic cells (orange). Right: annexin V only signal from wounded embryos. Both wounds are 10 min post-laser ablation. Scale bar, 10 µm. **e**,**f**, Fold change in annexin V labelling at wound edge (**e**) or individual cells (**f**) in DMSO (circles) or Fer-1 (squares) injected embryos. The asterisks indicate statistical significance (compared at 5 (**e**) or 2 (**f**) min intervals, Kruskal–Wallis tests with Dunn’s multiple comparisons tests; *P*_adj_ <0.05, *N* = 8 wounded embryos per drug and 24 cells per drug, respectively). The error bars are the mean ± s.e.m. **g**,**h**, Annexin V labelling of bona fide mammalian ferroptosis. **g**, Ferroptosis induced in Caco-2 intestinal epithelial cells involves annexin V labelling. Top: the ferroptosis inducer, ML162, triggered universal swelling and co-labelling with SYTOX (green) and annexin V (red, arrow). The boxed region is magnified (right). Bottom: ML162-induced ferroptosis was inhibited by Lip-1, preserving organoid integrity (left) and suppressing both annexin V (middle) and SYTOX (right) labelling. DIC, differential interference contrast. **h**, ML162-induced organoid ferroptosis is inhibited by liproxstatin-1. The graph shows a percentage of 3D structures with ten or more cells per structure labelled with SYTOX-Green, indicating cell death (*n* = 60 structures). Treatment with Lip-1 reduces necrosis induced by ML162. Source data

As observed in previous studies in the Zebrafish, lipid peroxidation also appeared to emanate outwards from the wound, through the surrounding healthy tissue (Fig. 3a)^32^. Furthermore, we established that lipid peroxidation preceded annexin V labelling during UV-induced, single-cell necrosis, through which we could strictly control the initiation of cell death (Fig. 2d,e). During the latter, we noted intense foci of lipid peroxidation in the immediate aftermath of UV irradiation (Fig. 2d). We speculated that these puncta might be mitochondria, the dysregulation of which are associated with ferroptosis^3,13^. We examined mitochondrial activity during UV-induced, single-cell necrosis using the mitochondrial dye, tetramethylrhodamine ethyl ester (TMRE), and observed immediate depolarization, preceding annexin V labelling (Extended Data Fig. 4e). Furthermore, mitochondrial depolarization was also evident within wounds generated through laser ablation and laser-free needle puncture, most notably within annexin V-positive necrotic cells (Extended Data Fig. 4f,g). Importantly, the sustained loss of TMRE signal was not due to photobleaching (Extended Data Fig. 4h). We, therefore, concluded that mitochondrial depolarization was a feature of this necrosis.

Next, we co-injected BODIPY^581/591^ C11 with established inhibitors of ferroptosis, ferrostatin-1 (Fer-1) and liproxstatin-1(Lip-1)^2,13^. In contrast to dimethyl sulfoxide (DMSO), both Lip-1 and Fer-1 suppressed wound-induced lipid peroxidation (Fig. 3a–c). Furthermore, Fer-1 suppressed UV-induced, single-cell necrosis, implying that lipid peroxidation is the causative agent of this cell death (Extended Data Fig. 4i). A key, negative regulator of lipid peroxidation in mammalian cells is the selenoprotein, GPX4. Interestingly, overexpression of the *Drosophila* homologue of GPX4, Phgpx, failed to supress UV-induced, single-cell necrosis (Extended Data Fig. 4i). However, Phgpx lacks the critical selenocysteine found in mammalian GPX4 and *phgpx* mutant flies are also adult viable and fertile, contrasting with the lethality of disrupting *gpx4* in mice^13,33,34^. Therefore, we conclude that *Drosophila* has evolved distinct, antioxidant defence mechanisms to protect itself from the accumulation of lipid peroxidation and subsequent ferroptosis.

**Extended Data Fig. 5:**
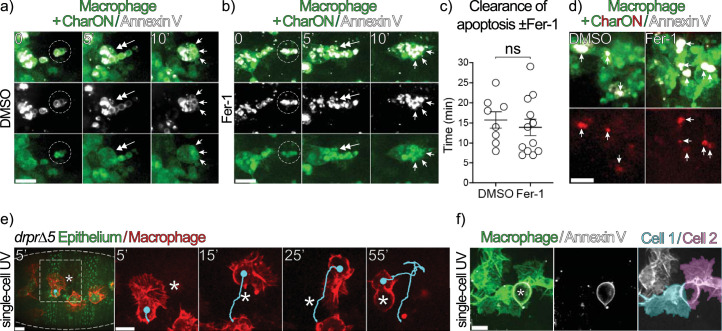
Macrophage-mediated clearance of apoptosis and ferroptotic necrosis. **5a–d**) Fer-1 does not perturb the clearance of developmental apoptosis. DMSO (control) or Fer-1 was co-injected with far-red Annexin V into embryos ubiquitously expressing CharON (dual biosensor of apoptosis and its clearance) and a macrophage-specific marker (GFP-moesin). The macrophage-mediated clearance of developmental apoptosis (embryonic stage 12–13) was then studied using 3-colour imaging. Note: Annexin V labelling implies corpse exposure to drugs pre-internalisation within a macrophage. (**a**, **b**) Macrophages (green) clearing of Annexin V (white) and CharON (green) labelled apoptotic corpses (dashed circle) in the presence of (**a**) DMSO or (**b**) Fer-1. In both cases, there is robust uptake (double headed arrows) leading to internalisation (barbed arrows). Time =minutes, scale bars =10 µm. (**c**) Time taken to engulf apoptotic debris (from first contact to internalisation) is not significantly different (ns) between DMSO or Fer-1 injected embryos (unpaired t-test, p ≥ 0.5, n ≥ 8 engulfment events from 3 embryos). (**d**) Fer-1 does not impair phagolysosome formation during clearance of apoptosis. (TOP) Clusters of macrophages (green) exhibiting internalised Annexin V (white) and CharON (green and red) labelled apoptotic corpses. (BOTTOM) Robust acidification (acid-activated fluorescence of phlorina component of CharON probe, red) of engulfed corpses (arrows) in both DMSO and Fer-1 injected embryos. Scale bar =10 µm. **5e**) Draper is required for the macrophage recruitment to, but not engagement of, single-cell ferroptotic necrosis. LEFT: Overview of ventral epithelium (GFP-moesin, green, outlined) of *drpr*∆^5^ mutant embryo, 5 minutes post-UV-induced, single-cell ferroptotic necrosis (asterisk). Boxed region highlights tracked macrophage (lifeact-mcherry, red, cell track shown in cyan). RIGHT: Adjacent panels show magnified, timelapse images of highlighted macrophage. In the first 10 minutes post-UV (during which control macrophages are rapidly recruited) the *drpr* mutant macrophage ignores single-cell ferroptotic necrosis. However, eventually after 30–60 minutes post-UV, the macrophage does successfully engage with the ferroptotic corpse. Note^1^: Far-red Annexin V not injected to exclude any interference with macrophage-corpse interaction. Note^2^: Slight shift in embryonic epithelium (and, therefore, the ferroptotic corpse) relative to tracked macrophage. Time = minutes, scale bar =10 µm. **5f**) Multi-macrophage envelopment of single-cell ferroptotic necrosis. UV-induced, single cell ferroptotic necrosis (asterisk) triggers rapid macrophage (lifeact-GFP, green) recruitment to the Annexin V (white) labelled ferroptotic corpse. Pseudo-colouring distinguishes the two macrophages involved in enveloping this ferroptotic corpse (shown 12 minutes post-UV). Scale bar =10 µm. Source data

To investigate how Fer-1 alters the pattern of necrosis during laser-induced injury, we injected the inhibitor together with SYTOX and annexin V. Following Fer-1 injection, annexin V labelling but not SYTOX uptake was strongly suppressed within the wound (Fig. 3d). Quantification of annexin V levels at the wound edge in general or at the membranes of individual, SYTOX-positive cells ringing the wound, confirmed Fer-1 blocked annexin V labelling during acute injury (Fig. 3e,f). While there was no evidence that Fer-1 suppressed necrosis during wounding (Extended Data Fig. 4j), the scale of insult caused by laser ablation makes cell survival unlikely. Instead, we propose that Fer-1 inhibition is specifically suppressing the ferroptotic programme during necrotic tissue damage. Importantly, Fer-1 had no effect on developmental apoptosis, its labelling with annexin V or its clearance (Extended Data Fig. 5a–c).

Finally, we investigated whether annexin V labelling was also a feature of mammalian ferroptosis. Caco-2 cells were cultured to form three-dimensional (3D) organoids and ferroptosis was induced by GPX4 inhibition using ML162. Ferroptosis induction was associated with pronounced cellular swelling and co-labelling with SYTOX and annexin V, which were strongly suppressed by Lip-1 (Fig. 3g,h and Supplementary Video 6). Furthermore, the extreme cellular swelling observed in both models is consistent with the established osmotic mechanism involved in ferroptosis^28^. We propose that the distinct ferroptotic necrosis we have revealed during acute injury represents in vivo *Drosophila* ferroptosis.

### Macrophages are actively recruited to ferroptotic necrosis

**Fig. 4:**
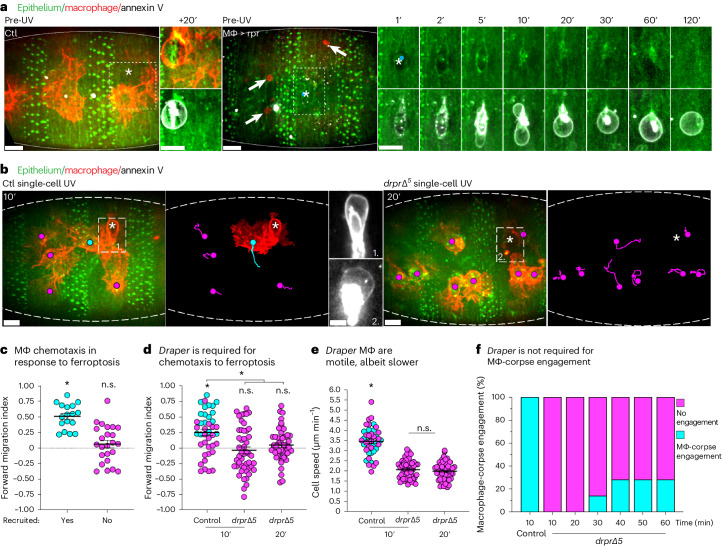
Macrophages are recruited to ferroptotic necrosis through Draper-dependent chemotaxis. **a**, Ferroptotic necrosis is actively extruded from the epithelium. Left: overview of control (Ctl) ventral embryonic epithelium (GFP-moesin, green, outlined) pre-UV irradiation. Following UV-induced, single-cell ferroptotic necrosis (asterisk), far-red annexin V (white)-labelled corpse is extruded from the epithelium and enveloped by a recruited macrophage (lifeact-mcherry, red). Middle: overview of ventral epithelium pre-UV irradiation in embryos where macrophages (Mϕ) have been ablated through pro-apoptotic reaper (rpr) expression (arrows highlight macrophage corpses). Right: UV-induced, single-cell ferroptotic necrosis (asterisk). Annexin V-labelled ferroptotic corpse is efficiently extruded from epithelium in the absence of macrophages. Time is indicated in minutes. Scale bars, 10 µm. **b**, Macrophages are recruited to single-cell ferroptotic necrosis. Overviews of control (left) or *drpr*∆^5^ mutant (right) ventral embryonic epithelium (GFP-moesin, green, outlined) 10 (control) or 20 (*drpr* mutant) min post-UV-induced, single-cell ferroptotic necrosis (asterisks). The macrophage (lifeact-mcherry, red) tracks and response are indicated by coloured dots (magenta, not recruited; cyan, recruited) ± cell tracks. *drpr* mutant macrophages are not recruited to ferroptotic necrosis even 20 min post-UV. Middle: magnified inserts of boxed regions in control (1) and *drpr* mutant (2) mutants, revealing the far-red annexin V (white)-labelled, ferroptotic corpses. Scale bars, 10 µm. **c**–**e**, Macrophages undergo Draper-dependent chemotaxis towards single-cell ferroptotic necrosis. All the data points are colour coded according to response to the ferroptotic corpse (cyan, recruited; magenta, not recruited). The error bars are the mean ± standard error of the mean (s.e.m.). (c) Macrophages recruited to single-cell ferroptotic necrosis exhibit significantly non-zero (asterisk, one sample *t*-test and Wilcoxon test, *P* < 0.0001) FMI, implying active chemotaxis. Non-responding macrophage FMI is random (not significantly (n.s.) different from zero, one sample *t*-test and Wilcoxon test, *P* > 0.05). Macrophages tracked over 10 min post-UV, *n* = 17 responding cells and 24 non-responding cells, *N* = 6 embryos). **d**, Significantly non-zero (asterisk, one sample *t*-test and Wilcoxon test, *P* < 0.0001) FMI exhibited in pooled control tracks, implying a general chemotactic response to single-cell ferroptotic necrosis. In contrast, no *drpr* mutant macrophages were recruited to single-cell ferroptotic necrosis within 10 or 20 min post-UV. Furthermore, no evidence of chemotactic response was exhibited by *drpr* mutant macrophages (FMI not significantly different (n.s.) from zero, one sample *t*-test and Wilcoxon test, *P* > 0.05). Control FMI is significantly higher than *drpr* FMI at both timepoints (asterisk, one-way ANOVA with Tukey’s multiple comparisons test, *P*_adj_ < 0.05). The controls include 41 cells and *N* = 6 embryos. The *drpr* mutants include 49 cells and *N* = 8 embryos. **e**, *draper* mutant macrophages are motile, albeit significantly slower than control macrophages (asterisk, one-way ANOVA with Tukey’s multiple comparisons test, *P*_adj_ < 0.0001) when tracked over 10 or 20 min post-UV. n.s., nonsignificant (*P* > 0.05). The controls include 41 cells and *N* = 6 embryos. The *drpr* mutants include 49 cells and *N* = 8 embryos. **f**, *draper* is not required for corpse engagement. The percentage of single-cell ferroptotic corpses engaged (cyan) or not engaged (magenta) by macrophages in control and *draper* mutant embryos are indicated at the timepoints. Source data

The ability to induce and visualize single-cell ferroptotic necrosis in vivo offered us an unprecedented opportunity to study how the surrounding tissue and immune system react when confronted with this cell death modality. For instance, it is unknown whether ferroptosis is recognized and actively extruded by the surrounding tissue as observed with apoptosis^35^. Given the rapid association with macrophages, we first determined whether these immune cells were required to remove ferroptotic necrosis from within the epithelium. To test this hypothesis, we utilized *Drosophila* genetics to ablate macrophages within the embryo and performed UV-induced, single-cell ferroptotic necrosis. In the absence of macrophages, we found that the epithelium was capable of recognizing and extruding single-cell ferroptotic necrosis (Fig. 4a). Instead, we postulated that macrophages were actively recruited to ferroptotic necrosis to clear this volatile cell death. We tracked all ventral macrophages (both responders and non-responders) in the immediate aftermath of UV-induced, single-cell ferroptotic necrosis (Fig. 4b). Macrophage recruitment to the extruded ferroptotic corpse was rapid, occurring within 10 min of UV irradiation (Supplementary Video 2). Quantification of the forward migration index (FMI) revealed that macrophages were recruited to single-cell ferroptotic necrosis via active chemotaxis, in contrast to the random migration of the non-responsive macrophages (Fig. 4c). Importantly, the strength of the chemotaxis exhibited by the responding macrophages was sufficient to significantly increase the FMI of all macrophages, an unbiased proof of chemotaxis in response to ferroptotic necrosis (Fig. 4d).

We sought to mechanistically dissect macrophage chemotaxis to ferroptotic corpses in vivo. During the inflammatory recruitment of macrophages to wounds, the fly CED-1 homologue, Draper, is required for sensing and chemotaxis of macrophages to tissue damage^22,36,37^. Given the involvement of the ferroptotic programme within these wounds, we investigated whether Draper was similarly required for macrophage recruitment to single-cell ferroptotic necrosis. Strikingly, *draper* mutant macrophages were unresponsive to single-cell ferroptotic necrosis (Fig. 4b). Furthermore, quantification of macrophage FMI confirmed there was no chemotactic response to these dying cells in the absence of *draper* (Fig. 4d). Despite *draper* mutant macrophages exhibiting significantly slower migration, there was no recruitment to single-cell ferroptotic necrosis when the macrophages were tracked over compensatory longer time periods (Fig. 4d,e). Occassionally, if imaged for approximately an hour, some *draper* mutant macrophages did eventually successfully engage with single-cell ferroptotic corpses (Fig. 4f and Extended Data Fig. 5e). Often in these cases, the macrophage would initially migrate past the dying cell, ignoring the corpse within the 10 min time frame control macrophages were recruited. In the absence of active chemotaxis, the eventual engagement evidently occurs randomly. Consistent with the known role of Draper in inflammation, we concluded that *draper* is required for chemotaxis to ferroptotic necrosis but not the engagement necessary for engulfment.

### Ferroptotic necrosis leads to frustrated phagocytosis

**Fig. 5:**
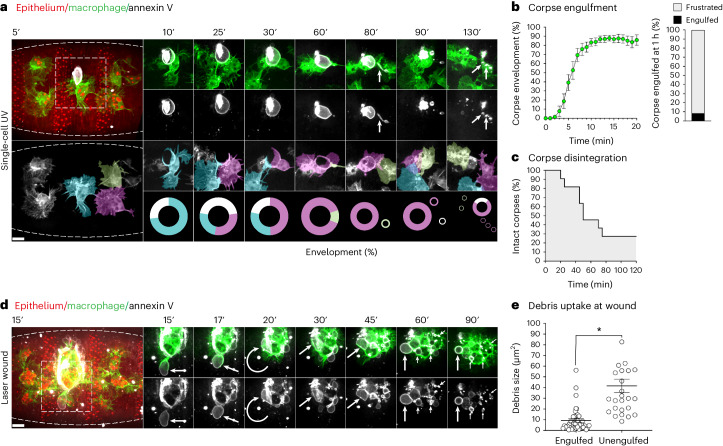
Ferroptotic necrosis leads to frustrated phagocytosis in macrophages. **a**, Top left: overview of ventral embryonic epithelium (mcherry-moesin, red, outlined) highlighting macrophage (lifeact-GFP, green) recruitment to far-red Annexin V (white) labelled, single-cell ferroptotic corpse (5 min post-UV). Bottom left: as above, excluding epithelium and annexin V. Pseudo-colouring highlights the three interacting macrophages. Right: timelapse images of macrophage (green) engagement with annexin V (white) labelled ferroptotic corpse. The arrows highlight corpse deformation and fragmentation (first and second rows); pseudo-colouring highlights interchange of macrophages at single-cell ferroptotic necrosis (third row); sector charts representing percentage of envelopment of corpse by macrophage (colour coded to match above) (fourth row). **b**, Macrophages engage but fail to fully envelop and engulf single-cell ferroptotic necrosis. Left: percentage of envelopment of single-cell ferroptotic necrosis by macrophages versus time post-UV. The error bars are the mean ± standard error of the mean (s.e.m.). *N* = 12 embryos. Right: bar chart highlighting per cent internalization of whole ferroptotic corpses (black) versus frustrated/stalled engulfment (grey) at 1 h post-UV. *N* = 12 embryos. **c**, Kaplan–Meier plot highlighting per cent intact ferroptotic corpses versus time post-UV. *N* = 11 embryos. **d**, Left: overview of laser-wounded ventral embryonic epithelium (mcherry-moesin, red) highlighting inflammatory macrophage (lifeact-GFP, green) recruitment to far-red annexin V (white)-labelled ferroptotic corpse shown 15 min post-wounding. Right: timelapse images corresponding to boxed region in overview showing macrophage engagement with an annexin V-labelled ferroptotic corpse (arrow) at the edge of wound. The curved arrow highlights corpse movement at wound edge. The barbed arrows indicate accumulation of annexin V-positive debris in macrophages. Time is indicated in minutes. Scale bar, 10 µm. **e**, Clearance of ferroptotic debris at wounds is limited to smaller particles. The mean size (µm^2^) of Annexin V-labelled debris, which is engulfed at the wound is significantly smaller (asterisk, Mann–Whitney test, *P* < 0.0001) than debris that is engaged but not engulfed after 30 min. *n* ≥ 26 particles per group, *N* = 5 wounded embryos. The error bars are the mean ± s.e.m. Source data

Recent work has highlighted that ferroptosis is poorly phagocytosed by immune cells in culture^38^. Therefore, we next investigated how macrophages clear ferroptotic necrosis in vivo. Control macrophages respond immediately to single-cell ferroptotic necrosis and envelop the extruded corpses within 10 min (Fig. 5a,b and Supplementary Video 2). However, despite this rapid engagement, the internalization of intact single-cell ferroptotic necrosis was extremely poor, occurring less than 10% of the time within 1 h (Fig. 5b). This is in contrast to their robust ability to rapidly engulf developmental apoptotic corpses within 20 min (Extended Data Fig. 5c). Instead, it was far more probable that the ferroptotic corpse was burst, only then allowing efficient clearance of the disintegrated debris (Fig. 5a and 5c). Even in the rare cases where a macrophage was seemingly successful in engulfing the necrotic cell intact, this was in effect achieved by tearing the corpse away from its epithelial tether, leaving a ‘scar’ of annexin V-positive debris (Extended Data Fig. 1c). While macrophage engagement of single-cell ferroptotic necrosis was swift, it was noted that envelopment of the entire corpse was rarely 100% (Fig. 5b). Furthermore, envelopment of the swollen necrotic cell often involved multiple macrophages (Fig. 5a, Extended Data Fig. 5f and Supplementary Video 7). Live imaging revealed that macrophages often ‘wrestled’ with each other in their attempts to envelop the corpse, and it was in these moments that rupture occurred. Competing macrophages would sometimes pull forcibly at exposed regions of the dying cell causing severe deformation and ultimately leading to abrupt disintegration (Fig. 5a and Supplementary Video 7). In contrast to the frustrated engulfment exhibited with the intact ferroptotic cell, disintegration allowed the immediate clearance of the annexin V-positive debris. Finally, we investigated how ferroptotic necrosis contributed to inflammation during acute injury by first live imaging the clearance of ferroptosis by macrophages in wounded embryos. Consistent with what we had observed at the single-cell level, we found inflammatory macrophages at wounds rapidly engaged with the ferroptotic necrosis extruded from the wound edge (Fig. 5d and Supplementary Video 8). However, these corpses were highly mobile, chaotically swinging around the edge of the wound, even while the macrophages attempted to engage them. Uptake and internalization of whole ferroptotic corpses was rarely seen, even 1 h post-wounding. Furthermore, over this time period, macrophages at the wound increasingly accumulated small, annexin V-positive puncta, indicative of corpse disintegration (Fig. 5d,e and Supplementary Video 8). This is in stark contrast to the rapid and efficient engulfment of the necrotic core within the centre of the wound^20^. Therefore, we conclude that the clearance of ferroptotic necrosis represents a phagocytic challenge to macrophages during acute injury.

### In vivo ferroptotic necrosis triggers inflammation

**Extended Data Fig. 6:**
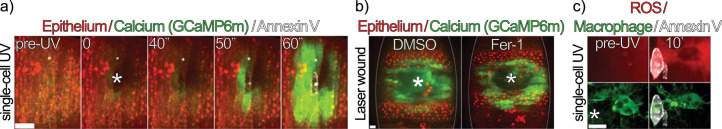
Single-cell ferroptotic necrosis recapitulates Drosophila inflammation. **6a**) Single-cell ferroptotic necrosis triggers Calcium signalling. 3-colour, *in vivo* imaging of Calcium (GCaMP6m, green) in epithelium (mcherry-moesin, red) surrounding UV-induced, single-cell ferroptotic necrosis (asterisk). UV-irradiation triggers immediate Calcium wave through epithelium, emanating from the Annexin V (white) labelled, ferroptotic corpse (arrow). Time = seconds, scale bar = 10 µm. **6b**) Fer-1 does not block wound-induced calcium signalling. Laser-wounding of ventral epithelium of embryo (mcherry-moesin) triggers immediate calcium wave (GCaMP6m, green) in embryos injected with either DMSO or Fer-1. Time = 30 s post-ablation, scale bars =5 µm. **6c**) ROS release during single-cell ferroptotic necrosis. 3-colour, *in vivo* imaging of ROS production during UV-induced, single-cell ferroptotic necrosis. Embryo co-injected with far-red Annexin V (white) and Amplex Red (ROS, red) followed by UV-irradiation of individual epithelial cell (asterisk). Annexin V labelled single-cell ferroptotic necrosis exhibits intracellular ROS generation and release, which envelops a proximal macrophage (GFP-moesin, green), coinciding with its recruitment. Time =10 minutes post-UV, scale bars = 10 µm. Note^1^: Distinct cell from Extended Data Fig. 2b. Note^2^: Non-homogenous ROS signal due to pooling of Amplex dye in interstitial cavities where macrophages reside. Source data

While the prominent release of damage associated molecular patterns (DAMPs) during ferroptosis implies it is a highly inflammatory form of cell death, its inability to trigger an adaptive response might suggest an inflammation-silencing activity^38^. Furthermore, the absence of direct in vivo imaging of ferroptosis has confounded attempts to test the contribution of ferroptosis to inflammation. We have demonstrated that macrophages are rapidly recruited to single-cell ferroptotic necrosis (Fig. 4b–d). Next, we investigated whether in vivo ferroptotic necrosis was indeed an inflammatory cell death and aimed to uncover its contribution to acute inflammation. The earliest known inflammatory mediator triggered in the wounded *Drosophila* embryo is a calcium wave emanating out from the wound, which in turn triggers ROS production^29,30,39^. Utilizing single-cell ferroptotic necrosis and three-colour imaging, we demonstrated that ferroptotic necrosis alone is able to trigger a calcium wave through the surrounding tissue (Extended Data Fig. 6a). Fer-1 suppresses single-cell ferroptotic necrosis, constraining our ability to interrogate the precise role of the ferroptotic programme in calcium signalling (Extended Data Fig. 4i). However, a robust calcium wave was observed emanating from laser-induced wounds in embryos injected with Fer-1, implying that ferroptotic necrosis is sufficient but not necessary for this earliest of inflammatory signals (Extended Data Fig. 6b). We have demonstrated that single-cell ferroptotic necrosis triggers intracellular ROS generation, but we also observed release of ROS from the ferroptotic corpse, which subsequently surrounded the neighbouring macrophages (Extended Data Figs. 4b and 6c). Combined with the fact that the recruitment of macrophages to a ferroptotic corpse requires Draper (Fig. 4b,d), ferroptotic necrosis appears to recapitulate everything currently known about wounding and the inflammatory response in the *Drosophila* embryo^40^. Furthermore, an active role for ferroptotic necrosis within acute injury is in agreement with mathematical modelling that predicted the presence of a distinct form of necrosis, which is required to perpetuate calcium signalling in wounded *Drosophila* pupae^41,42^.

**Fig. 6:**
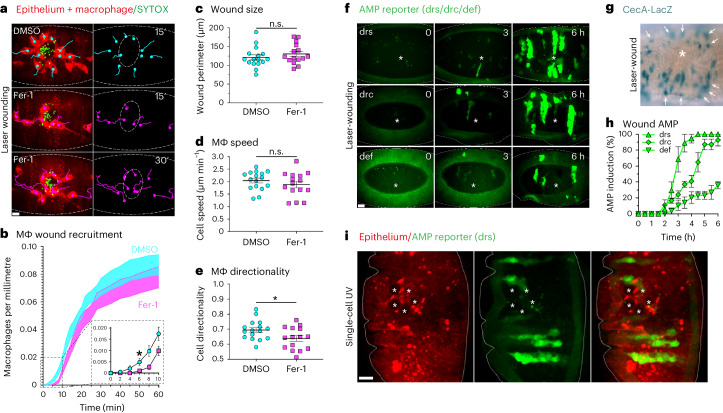
The role of ferroptotic necrosis in inflammatory tissue damage. **a**, Suppression of ferroptotic necrosis during acute injury delays the inflammatory recruitment of macrophages. Ventral epithelium (mcherry-moesin, red, outlined) of embryos co-injected with SYTOX (necrotic dye, green) and either DMSO (control) or Fer-1. Left: following laser wounding, macrophages (mcherry, red) were tracked (DMSO, cyan tracks; Fer-1, magenta tracks) during their inflammatory recruitment to SYTOX-labelled tissue damage. Right: tracks only with wound outlined by dashed oval. Time is indicated in minutes. Scale bar, 10 µm. **b**, The number (accumulating total) of recruited macrophages normalized to wound perimeter (µm) 1 h after wounding. The shaded areas delineate the mean ± standard error of the mean (s.e.m.) (DMSO injected controls, cyan; Fer-1, magenta). The range 0–10 min is enlarged in insert (dashed boxes), highlighting delayed inflammatory recruitment of macrophages, reaching significance at 6 min post-wounding (asterisk, Kruskal–Wallis test with Dunn’s multiple comparisons test; *P*_adj_ = 0.0495, *N* ≥ 15 wounded embryos/injection regime). The error bars are the mean ± s.e.m. **c**–**e**, Quantification of effect of Fer-1 on wounding revealed no significant difference (n.s.) in wound size (perimeter, µm) at 5 min post-wounding (**c**) and n.s. in macrophage speed during inflammatory recruitment (unpaired *t*-tests, *P* > 0.05) (**d**). Instead, macrophage directionality was significantly reduced (asterisk, unpaired *t*-test, *P* = 0.032) by Fer-1 (**e**). The embryo means are plotted (*N* ≥ 15 wounded embryos per injection regime). The error bars are the mean ± s.e.m. **f**–**h**, Sterile laser wounding triggers local AMP production. Expression of GFP–AMP reporters (green) in embryo (outlined) at indicated timepoints (hours) after laser-wounding (asterisks) (**f**). CecA-Lacz staining (turquoise) of laser-wounded (asterisk) embryo (**g**). The arrows highlight CecA-Lacz-positive epithelium cells surrounding wound. Percentage of embryos exhibiting GFP–AMP expression hours after sterile laser wounding (**h**). The error bars are the mean ± s.e.m. drs, drosomycin; drc, drosocin; def, defensin; cecA, cecropinA. Scale bars, 10 µm. **i**, Sterile ferroptotic necrosis is sufficient to trigger epithelial AMP production. A cluster of ferroptotic necrosis induced in embryo (outlined) by UV irradiation (asterisks) of five epithelial cells (mcherry-moesin, red) is shown. A total of 8 h after UV irradiation, the expression of a GFP–AMP reporter (drs) was detected in the epithelium surrounding ferroptotic necrosis. Scale bar, 10 µm. Source data

Consistent with ferroptotic necrosis being sufficient to stimulate the earliest known inflammatory mediators, we found that injection of Fer-1 suppressed macrophage recruitment during the initial stages of inflammation (Fig. 6a,b and Supplementary Video 9). While this effect was subtle, it is consistent with our finding that Fer-1 does not prevent necrotic tissue damage within the wound per se (Extended Data Fig. 4j). Instead, we propose that the inhibition of the ferroptotic programme during necrotic tissue damage slows the release of DAMPs, which delays inflammation rather than suppressing it. This result was not due to any difference in wound size or change in macrophage speed (Fig. 6c,d). Instead, we found Fer-1 specifically reduced the directionality of macrophages during their recruitment to the wound edge, supporting the conclusion that ferroptotic necrosis enhances the inflammatory recruitment of macrophages during acute injury (Fig. 6e and Supplementary Video 9). Lastly, it has been previously observed that sterile, laser-induced wounding triggers a local production of various anti-microbial peptides (AMPs) within the wounded epithelium in anticipation of pathogen entry and subsequent infection^43^. We confirmed this through the use of different reporter lines for a number of AMPs, the expression of which were detected in the epithelium surrounding the wound, several hours after injury (Fig. 6f–h)^44,45^. We made use of this anti-microbial response as a further, functional readout of inflammation by combining UV-induced ferroptotic necrosis with an AMP reporter^44^. We found that a small cluster of sterile, UV-induced ferroptotic necrosis was sufficient to trigger AMP production in the surrounding epithelium (Fig. 6i).

We concluded that ferroptotic necrosis actively contributes to inflammation through triggering inflammatory mediators, enhancing macrophage recruitment and stimulating AMP production in advance of expected pathogen entry.

## Discussion

Here, through cutting-edge, three-colour, in vivo imaging, we have visualized necrosis during tissue damage and revealed the presence of a ferroptosis-like necrosis as an integral component of acute injury. While ferroptosis is the ultimate outcome of overwhelming lipid peroxidation, the ferroptosis-like cell death we have identified here in *Drosophila* operates independently of the upstream negative regulator, GPX4. This suggests the fly has evolved a distinct antioxidant defence system to counteract accumulating lipid peroxidation. Interestingly, *Drosophila* cell membranes are highly monosaturated, providing one explanation as to how its cells might protect themselves against lipid peroxidation. Nevertheless, while this necrosis is dependent on lipid peroxidation, we suggest that this cell death should be considered ‘dysregulated ferroptosis’, pending the identification of the regulators of ferroptosis in *Drosophila*.

In this study, we confirm that ferroptotic necrosis is indeed an inflammatory form of cell death, contributing to the recruitment of immune cells during wounding and the triggering of the tissues innate anti-microbial defences. However, we demonstrate that ferroptotic necrosis is inherently challenging to clear by macrophages, leading to frustrated engulfment and frequent corpse disintegration. For this reason, it might seem counterintuitive that organisms would elicit the ferroptotic programme during acute injury. However, the toxic biological pathways that are spiralling out of control during ferroptosis are probably beyond what can be channelled into an evolutionary beneficial outcome and must instead be managed by the immune system as best it can. Inflammation is inherently a compromise, with many undesirable outcomes as evidenced by the fact that the influx of innate immune cells during acute injury can cause considerable collateral damage^46^.

Moreover, the difficulty faced by macrophages confronted with the clearance of ferroptotic necrosis might help address the paradox of how the lytic and highly inflammatory ferroptosis is seemingly so poor at promoting antigen presentation and, therefore, triggering an adaptive immune response^38^. In addition to impaired engulfment hindering antigen presentation, it is possible that ferroptosis actively suppresses antigen presentation to prevent autoimmunity arising from intracellular content release. The spillage of intracellular contents resulting from frustrated phagocytosis is undoubtedly an unintended consequence of the clearance of ferroptotic necrosis. Furthermore, given the toxic nature of the peroxidized lipid underlying ferroptosis, the release of this volatile debris is also probable to be a negative aspect of corpse disintegration. As such, ferroptosis probably has conflicting roles in acute injury, raising important considerations for treating organ damage and harnessing ferroptosis for enhancing cancer therapy. In the case of the former, our findings support a key role of the ferroptotic programme in tissue damage, which will probably be exacerbated by the inefficient clearance of this necrosis. Furthermore, the macrophage-mediated disintegration of ferroptotic necrosis we have documented risks provoking autoimmunity. On the other hand, while it appears ferroptosis does not mobilize a robust adaptive immune response against cancer, we show its highly inflammatory nature triggers a strong innate immune response, which might be beneficially targeted at tumours. Ultimately, the ability to induce and visualize ferroptotic necrosis within the genetically tractable fly offers a powerful model with which to dissect the consequences of ferroptosis to organismal physiology and pathology.

## Methods

### *Drosophila* stocks

For labelling of embryonic macrophages with UAS-driven constructs, *singedGAL4* was used (*sn-gal4*)^47^. For the labelling of the embryonic epithelium with UAS-driven constructs, *e22cGAL4* was used (*e22c-gal4*)^48^. The ubiquitous expression of the UAS-CharON biosensor throughout the embryo was achieved using *daughterlessGAL4* (*da-gal4*)^49^. GAL4-independent labelling of the embryonic macrophages was achieved using *srp-gma* or *srp-3xmcherry*^50^. The GAL4-independent labelling of the embryonic epithelium was achieved using *sqh-mcherry-moesin* and *ubi-gma*^51,52^. The following UAS constructs were used in this study: *uas-lifeact-gfp, uas-lifeact-mcherry*, *uas-mcherry-moesin*, *uas-reaper*, *uas-gc3ai*, *uas-charon* and *uas-gcamp6m* (refs. ^20,47,53–57^). The *uas-phgpx* line was generated for this study (synthesized and cloned into pUASt by GeneArt, Thermo Fisher Scientific, with transgenics performed by BestGene). The AMP reporters used were *drs-gfp-drs*, *drc-gfp*, *def-gfp-drs* and *ceca1-lacz*^44,45,58^. The amorphic null alleles used as part of this study were: *drpr∆*^*5*^*, ∆h99, dark*^*cd4*^ and *dronc∆*^*51*^ (homozygote embryos used in all cases)^59–62^. A list of the genotypes of all fly lines used in this study can be found in Supplementary Table 1. The majority of all fly lines used as part of this work were derived from those ordered through the Bloomington Stock Centre (University of Indiana (NIH P40OD018537)). FlyBase was also used extensively for genetic and molecular information^63^.

### Live imaging

All live imaging was performed using an inverted spinning disc confocal microscope (PerkinElmer Ultraview) using a plan-apochromat 63× objective with a NA of 1.4 and a Hamamatsu C9100-14 camera. The acquisition software used was Volocity (PerkinElmer). Images of different channels were acquired sequentially, changing the filters between each *Z*-stack to eliminate bleed through between channels during two- to three-colour imaging. UV-induced, single-cell necrosis was induced using the FRAPPA unit (PhotoKinesis module) on the UltraVIEW spinning disc system, allowing targeted irradiation of individual epithelial cells (100% 405 nm laser, 300 cycles of 100–200 ms bleaches (~40–60 s total), using the crosshair at the smallest spot size). For the fluorescence recovery after photobleaching (FRAP) of TMRE, photobleaching was performed as above, except the 561 nm laser was used. Epithelial wounds were generated using laser ablation (nitrogen-pumped micropoint ablation laser tuned to 435 nm, Andor Technologies). For needle puncture, a broken Femto tip needle was stabbed into the ventral face embryos using a FemtoJet injectman rig (Eppendorf).

For embryonic imaging, caged flies were left to lay over night at 25 °C, and the resulting embryos were collected in cell strainers (Falcon). The embryos were then dechorionated with bleach (Jangro) and washed repeatedly with water, and the embryos were developmentally staged based on gut morphology (all embryos imaged at stage 15 unless otherwise stated in the figure legends). The embryos were then mounted ventral side up on scotch tape between a glass slide and a supported coverslip in droplets of VOLTALEF oil. The slide was then inverted for imaging of the ventral epithelium of the embryo. *Z*-stacks (10–20 µm × 0.5–1 µm slices) of the ventral macrophages were then acquired on the UltraVIEW spinning disc system.

For the microinjection of dyes and inhibitors, embryos were dechorionated, washed and mounted as normal before being dehydrated in a sealed box with silica beads for ~15–30 mins at 25 °C. A droplet of VOLTALEF was added to each embryo before anterior injection into the intervitelline space surrounding the head of the embryo. Microinjection was performed using an InjectMan4 microinjector (Eppendorf) combined with a FemtoJet injectman rig (Eppendorf) fitted with Femto tips (Eppendorf). A coverslip was sealed on top and imaging undertaken immediately (dyes) or after a 10-min incubation (inhibitors). Annexin V–Alexa Fluor 647 (Molecular probes, Life Technologies) was injected neat when used alone. For co-labelling with SYTOX-Green (Invitrogen, Thermo Fisher Scientific), a 1/10 dilution of SYTOX in phosphate-buffered saline (PBS) (3 µM) was mixed with neat annexin V-647 at a 1:9 ratio. For osmoprotectant experiments, a 1/5 dilution of SYTOX in PBS (6 µM) was mixed with neat annexin V-647 and 345 mM solution of either sucrose (Merck), PEG1450 (Merck) or PEG3350 (Merck) at a 1:5:4 ratio. To specifically test the labelling of developmental apoptosis in the presence of PEG, the same mixtures were injected directly into the embryonic central nervous system (CNS). To image the ROS, Amplex UltraRed Reagent (Invitrogen, Thermo Fisher Scientific) was dissolved in DMSO (10 mM) and mixed with neat annexin V-647 at 1:9 ratio. For ratiometric imaging of lipid peroxidation, BODIPY 581/591 C11 dye (Molecular probes, Thermo Fisher Scientific) was dissolved in DMSO (30 mg ml^−1^) and mixed with neat annexin V at 1.5:10 ratio. Alternatively, BODIPY 581/591 C11 dye was mixed with PBS and either neat DMSO, 10 mg ml^−1^Fer-1 (Merck) or 10 mg ml^−1^liproxstatin-1 (Merck) at a 1.5:6:2.5 ratio. For labelling 4-HNE, a rabbit anti-4-HNE polyclonal antibody, conjugated with Alexa Fluor 488 (bs-6313R-A488, Bioss Antibodies) was diluted 1/10 in PBS and mixed with annexin V-647 at a 1:1 ratio before injection. For imaging necrotic tissue damage in presence of inhibitors, a 1/5 dilution of SYTOX-Green in PBS (6 µM) was mixed with neat annexin V-647 and either neat DMSO or Fer-1 at a 0.5:7:2.5 ratio. To specifically test the labelling of developmental apoptosis in the presence of Fer-1, the same mixtures were injected directly into the embryonic CNS. To suppress single-cell ferroptotic necrosis, DMSO or Fer-1 was injected at a ratio of 0.5:2.5:7. For imaging the clearance of CharON expressing apoptosis in presence of Fer-1, either DMSO or 10 mg ml^−1^Fer-1 were mixed with annexin V-647 at a 2.5:7.5 ratio and injected into the embryonic haemocoel. For imaging mitochondrial polarization, TMRE dye (Invitrogen, Thermo Fisher Scientific) was dissolved in DMSO (25 mg ml^−1^) and either diluted 1/10 with PBS and injected or mixed with neat annexin V-647 at a 1:9 ratio before injection.

### Immunohistochemistry

Dechorionated embryos were rapidly fixed in mix of hepatane (Merck) and 37% formalin (Merck) at 1:1 ratio in glass vials. After 30 s of vigorous shaking by hand and a further 2 min on rotating wheel, the fix was removed and replaced with 100% methanol (Merck). After vigourously shaking by hand for 30 s, the embryos were transferred in methanol to a 1.5 ml tube (Eppendorf) and washed repeatedly with methanol. The embryos were then washed repeatedly in PBS–Triton X (0.3%, Merck)–BSA (0.5%, Merck) solution before overnight incubation at 4 °C with rabbit anti-c-dcp-1 primary antibody (Cell Signalling Technology), diluted 1:500 in PBS–Triton X (0.3%)–BSA (0.5%) solution. The following day, the embryos were washed with PBS–Triton X (0.3%)–BSA (0.5%) solution and blocked using 2% horse serum (Merck) diluted in PBS–Triton X (0.3%)–BSA (0.5%) solution for 30 min on a rotating wheel. After repeated washing with PBS–Triton X (0.3%)–BSA (0.5%) solution, the embryos were incubated for 1 h at room temperature in goat anti-rabbit-AlexaFluor 488 (Thermo Fisher Scientific), diluted 1:200 in PBS–Triton X (0.3%)–BSA (0.5%) solution. After repeated washing in PBS–Triton X (0.3%)–BSA (0.5%) solution, the embryos were transferred to Vectashield mounting medium (Vector Labs) and mounted as described above. For imaging fixed embryos, a Zeiss LSM 880 confocal microscope was used, utilizing an Airyscan detector and a plan-apochromat 40× objective with a NA of 1.3. The acquisition software used was Zen Black (Zeiss). The hard fix preserved the fluorescence of the mcherry-labelled macrophages and epithelium, allowing detection in the absence of immunostaining. For LacZ staining, wounded embryos were fixed in glutaraldehyde-saturated heptane and stained overnight with 5-bromo-4-chloro-3-indolyl-β-d-galactoside.

### Mammalian cell culture

Caco-2 cells (ATCC) were cultured in Eagle’s minimum essential medium (ATCC) supplemented with 20% foetal bovine serum (Sigma Aldrich) and 1% penicillin–streptomycin (Corning) at 37 °C in 5% CO_2_. The on-top culture method was used to grow the 3D structures; the cells were trypsinized and resuspended (10^4^ cells per mililitre) in media with 2% Matrigel (Fisher Scientific). A total of 400 μl of suspension was plated in each well on a 48-well plate precoated with 30 μl of Matrigel. To induce ferroptosis, 3D cultures were treated with 4 µM ML162 (Selleckchem) and incubated with SYTOX-Green (Invitrogen, 15 nM) and annexin V CF 568 conjugate (Biotium, 1.25 µg ml^−1^) for 24 h. To inhibit organoid ferroptosis, 4 µM liproxstatin-1 (Selleckchem.com) was added along with the ML162. For imaging, the cells were incubated in an environmental chamber at 37 °C and 5% CO_2_, and fluorescence and differential interference contrast images were acquired using a Nikon Ti-E inverted microscope attached to a CoolSNAP CCD (charge-coupled device) camera (Photometrics) and NIS Elements software (Nikon).

### Image analysis

With the exception of the 3D renderings (where Arivis (Arivis AG) was used), ImageJ (NIH) was used for the processing and analysis of all images. All the analysed and presented images are *z*-projections (all spinning disc images are maximum *z*-projections and all Zeiss LSM 880 images are average *z*-projections). Excessive noise was removed from presented *z*-projected images using the ‘despeckle’ tool in ImageJ, otherwise only the brightness and contrast was adjusted. All analyses were performed on unprocessed, *z*-projected images.

For the quantification of Annexin V signal at the wound edge, a line with 12 pixel (2 µm) width was drawn around the mcherry-moesin-labelled wound edge using the ‘segmented line’ tool in ImageJ, and the mean signal under the area of the line was calculated using the ‘Measure’ function. The background was subtracted (the mean signal within a rectangle which spanned the bottom of the frame and excluded the embryo itself). Fold change in the signal was then calculated compared with the pre-wound image (using the wound outline from the subsequent, post-wound image). Annexin V labelling of individual cells was quantified as above but using a line with a 5 pixel (0.8 µm) width to outline mcherry-labelled, SYTOX-positive cells. Similarly, individual UV-irradiated, mcherry-moesin and annexin V-labelled epithelial cells were outlined using the ‘polygon selection’ tool, and area and circularity were calculated using the ‘Measure tool’. To quantify SYTOX uptake in these same wounds and cells, the mean intensity of the SYTOX signal was either measured within the area of the wound or in a 20 pixel (3.36 µm) diameter circle around the nuclei of individual cells, respectively, using the measure function in ImageJ. A background (mean signal within a rectangle spanning the bottom of the frame) subtraction was performed and the values were normalized to the signal in the pre-wound image. The total number of SYTOX-positive nuclei versus the number of annexin V and SYTOX co-labelled cells present in wounds over time was derived by manual counting. For the quantification of the osmprotectant experiments, the uptake of SYTOX was measured by drawing a 30 pixel (5 µm) diameter circle around the nuclei of irradiated cells using the ‘oval selection’ tool in ImageJ, and the mean signal within the area was calculated using the ‘Measure’ tool. Alternatively, annexin V uptake was measured by outlining the mcherry-labelled epithelial cell using the ‘Polygon selection’ tool, and the mean signal within the area was calculated. In both cases, the background was subtracted (the mean signal within a rectangle which spanned the bottom of the frame and excluded the embryo itself, for each respective channel). The fold change in the signal was then calculated compared to the pre-UV image.

For the quantification of cleaved-caspase staining of wounds, 2 µm *z*-projections of either macrophage-enveloped cells around the wound edge or c-DCP-1-positive apoptotic cells within the CNS were analysed, with the cells outlined using the ‘Polygon selection’ tool in ImageJ, and the mean signal intensity was calculated therein. These values were then normalized to background staining of the embryo (mean intensity of a square lacking any developmental apoptosis or wound).

For the ratiometric analysis of cellular lipid peroxidation using the BODIPY^581/591^ C11, individual cells either in direct contact with the wound edge or three cell rows back (controls) were outlined using the ‘freehand’ selection tool and the mean signal from both channels (that is lipid and lipid^ox^) within the area was measured. The lipid^ox^/lipid ratio was calculated, and fold change relative to the pre-wound image was plotted. This analytic approach was also undertaken to measure lipid peroxidation and annexin V labelling during UV-induced, single-cell necrosis (and corresponding control cells). To measure lipid peroxidation within the wound in presence of inhibitors, the entire wound was outlined using the ‘oval selection’ tool in ImageJ, and the mean signal from both channels (that is lipid and lipid^ox^) and the lipid^ox^/lipid ratio was calculated as above. For the pre-wound image, lipid and lipid^ox^ signal within the entire outlined embryo was measured, with the former plotted as an injection control. To quantify 4-HNE accumulation in wounds, an oval was fitted around the +90 min wound in ImageJ using the epithelial mcherry-moesin to delineate the wound edge. The mean anti-4-HNE signal within the oval was then calculated using the ‘measure’ function. This same oval was used to derive the mean signal at 0 min and the pre-wound values, the latter of which were used for a background subtraction.

For analysis of ferroptosis in 3D organoids, structures with ten or more SYTOX-positive cells by 24 h after the addition of ML162 were scored as necrotic.

For quantification of chemotaxis to UV-induced, single-cell necrosis, annexin V was not injected in case this perturbed macrophage-corpse recruitment or engagement. Instead, single-cell ferroptotic necrosis was visualized through epithelial cell swelling and extrusion using the mcherry-moesin label. The resulting images were rotated in ImageJ so the tracked macrophage (responder or otherwise) was horizontal to the irradiated cell at timepoint zero, with the macrophage at the line origin. The macrophage was then tracked using the ‘manual tracking’ tool in ImageJ over 10–20 min post-UV. These tracks were then imported into the ‘chemotaxis tool’ plugin and cell speed and FMI (a direct measure of chemotaxis wherein 0 is random migration and >0 is positive chemotaxis), along the *X* axis were derived. Responding cells were judged to be those that reached the irradiated cell within 10 or 20 min, respectively. To quantify corpse engagement, macrophage contact via the formation of a large, lifeact-mcherry positive phagocytic cup was scored as engagement. Percentage envelopment was quantified by measuring the circumference of the annexin V-labelled corpse over time using the ‘segmented line’ and ‘Measure’ tools in ImageJ. The length of the corresponding phagocytic cup extended by the macrophage around the corpse was similarly measured, and a percentage was derived. Corpse engulfment was judged to have occurred when a lifeact green fluorescent protein (GFP)-labelled macrophage was found to complete enclose the corpse (confirmed in unprojected *Z*-stack) and by the subsequent movement of the macrophage away from the site of engulfment. Corpse disintegration was judged to have occurred when the Annexin V-labelled corpse dramatically decreased in size and broke into multiple discrete particles. The particle uptake at wounds was quantified by identifying annexin V-positive debris, which was engaged by lifeact-GFP-labelled macrophages (that is, via observed phagocytic cup formation). The size of this debris (µm^2^) was then measured in ImageJ either at the point of uptake or, if not detectably internalized within a macrophage, at the end of the 30-min period analysed to quantify the clearance of CharON labelled, developmental apoptosis. The time taken for Casp-GFP and Annexin V co-labelled debris to be engulfed (from initial macrophage-corpse contact to fully internalized) was determined.

The inflammatory macrophage recruitment to wounds was analysed by tracking macrophages using the ‘manual tracking’ tool in ImageJ and scored for when they first made contact with the wound edge/mass of macrophages already recruited to the wound. These tracks were imported into the ‘chemotaxis tool’ in ImageJ to calculate the cell speed and directionality of recruited macrophages (FMI was not calculated for wounding data due to the sheer size of the wounds, which, therefore, lacked a definitive point with which to track macrophages to). To calculate wound size, the mcherry-moesin-labelled wound edge was outlined using the ‘segmented line’ tool in ImageJ, and the wound perimeter was calculated using the ‘Measure’ function macrophage recruitment was normalized to wound perimeter, as is best practice.

AMP-reporter expression in response to wounding was analysed by scoring wounded embryos for time taken for first detectable expression of reporter fluorescence (compared with non-wounded controls).

### Statistics and reproducibility

The specific statistical tests carried out, and their results are reported in the relevant figure legends. The individual *P* values in full are reported in Supplementary Table 2. Prism (GraphPad) was used for all statistical analysis. In all cases, the normality of every dataset was first tested using Shapiro–Wilk and Kolmogorov–Smirnov tests. Parametric tests (for example, *t*-test or analysis of variance (ANOVA)) were performed only when normality of all data within the dataset had been confirmed; otherwise, non-parametric tests were used (for example, Kruskal–Wallis tests). All *t*-tests were two sided. When analysing three or more groups within a dataset, an appropriate ANOVA or Kruskal–Wallis test was performed. Multiple comparison follow-up tests were used as reported in the relevant figure legends and a multiplicity adjusted *P* value (*P*_adj_) were derived. Each embryo was imaged individually and, therefore, represents an independent repeat (*N*, reported in figure legends). No statistical method was used to pre-determine sample size. No data were excluded from the analyses. The experiments were not randomized. The investigators were not blinded to allocation during experiments and outcome assessment.

### Reporting summary

Further information on research design is available in the Nature Portfolio Reporting Summary linked to this article.

## Online content

Any methods, additional references, Nature Portfolio reporting summaries, source data, extended data, supplementary information, acknowledgements, peer review information; details of author contributions and competing interests; and statements of data and code availability are available at 10.1038/s41556-024-01450-7.

## Supplementary information

Reporting Summary

Supplementary Information Supplementary Tables 1 and 2.

Supplementary Video 1 **Three-colour imaging of cell death during laser wounding of** ***Drosophila*** **embryo**. Laser ablation (asterisk) of the ventral epithelium (top right, mcherry-moesin, red) results in the inflammatory recruitment of macrophages (bottom left, lifeact-GFP, green) to the site of tissue damage. Far-red annexin V (top right, white) specifically labels extruded corpses around the edge of the wound. The first frame (−00:30) shows the embryo pre-wound. The embryo is outlined in white, the time is shown in minutes:seconds and the scale bar represents 10 µm. This video relates to Fig. 1b.

Supplementary Video 2 **Macrophages rapidly envelop UV-induced, single-cell death**. UV irradiation of an individual cell of the *Drosophila* embryonic epithelium (mcherry-moesin, not displayed) results in labelling with far-red annexin V (white) and extrusion. Macrophages (lifeact-GFP, green) are rapidly recruited and eventually engulf the swollen corpse. Left: maximum *z*-projections. Right: 3D renderings (generated using Arivis software). The first frame (pre-UV) shows the cell before the UV irradiation. The time is shown in minutes:seconds, and the scale bar represents 10 µm. This video relates to Extended data Fig. 1b.

Supplementary Video 3 **Three-colour imaging of a laser-ablated embryonic epithelium reveals a distinct pattern of necrosis within the wound**. Laser wounding triggers the inflammatory recruitment of macrophages (mcherry, red) to the wound. The necrotic core of the wound is strongly labelled by SYTOX (necrotic dye, green) but weakly labelled by far-red annexin V (white). In contrast, the necrotic cells ringing the edge of the wound are distinctly co-labelled with both SYTOX and annexin V. The dashed box magnified in the underlying panels. The first frame (pre-wound) shows the embryo before laser ablation. The time is shown in minutes:seconds, and the scale bars represent 10 µm. This video relates to Fig. 1d.

Supplementary Video 4 **UV-induced, single-cell death is necrotic**. UV irradiation of an individual cell of the *Drosophila* embryonic epithelium (mcherry-moesin, red) results in co-labelling of the corpse with both far-red annexin V (white) and SYTOX (necrotic dye, green). A macrophage (mcherry, red) is rapidly recruited and envelops the labelled corpse. The first frame (−00:45) shows the embryo pre-UV. The time is shown in minutes:seconds, and the scale bar represents 10 µm. The video relates to Extended data Fig. 1i.

Supplementary Video 5 **In vivo** **lipid peroxidation during wounding of the** ***Drosophila*** **embryonic epithelium**. Microinjection of BODIPY^581/591^ C11 into *Drosophila* embryos labels the membrane in red and shifts to green in the presence of lipid peroxidation. The circled cell tracked in the aftermath of laser ablation (magnified in adjacent panels) exhibits intense lipid peroxidation. The first frame (pre-wound) shows the embryo before laser ablation. The time is shown in minutes:seconds, and the scale bars represent 10 µm (left) and 2.5 µm (right), respectively. This video relates to Fig. 2b (distinct embryo).

Supplementary Video 6 **Mammalian ferroptosis co-labels with SYTOX and annexin V**. Timelapse microscopy of Caco-2 3D organoids treated with ML162 in the absence (top) or presence (bottom) of liproxstatin-1. DIC (left) and annexin V and SYTOX (right) images are shown. ML162 induces prominent cell swelling and annexin V labelling within ferroptotic 3D organoid, which is blocked by liproxstatin-1. The time is shown in minutes. This video relates to Fig. 3g, h.

Supplementary Video 7 **Macrophages ‘struggle’ to engulf ferroptotic corpses**. UV irradiation of an individual cell of the *Drosophila* embryonic epithelium (mcherry-moesin, not displayed) results in labelling with far-red Annexin V (white) and extrusion. Macrophages (lifeact-GFP, green) are rapidly recruited, but ‘struggle’ to envelop and engulf swollen corpse. Eventually, the involvement of multiple macrophages leads to bursting of the feroptotic corpse. Maximum z-projections (left) and 3D renderings (right) (generated using Arivis software) are shown. The first frame (pre-UV) shows the cell before the UV irradiation. The time is shown in hours:minutes:seconds, and the scale bar represents 10 µm. This video relates to Fig. 5a.

Supplementary Video 8 **Large ferroptotic corpses are challenging to clear during inflammation and wound healing**. The swollen, annexin V (white) labelled ferroptotic coprses generated by laser wounding (asterisk) of the *Drosophila* embryo area are rapidly engaged by macrophages (lifeact-GFP, green) as part of inflammation. However, uptake of large, intact corpses is minimal, and instead, macrophages at the wound accumulate smaller, annexin V-positive puncta. The time is shown in minutes, and the scale bar represents 10 µm. This video relates to Fig. 5d.

Supplementary Video 9 **Inhibition of ferroptotic necrosis impairs macrophage recruitment during inflammation**. *Drosophila* embryos were co-injected with SYTOX (necrotic dye, green) and either DMSO (control) or Fer-1. Following laser ablation of the ventral epithelium (mcherry-moesin, red, outlined), macrophages (mcherry, red) were tracked during their inflammatory recruitment to the wound (cyan tracks, DMSO; magenta tracks, Fer-1). Fer-1 impairs inefficient macrophage recruitment to necrotic tissue damage. The time is shown in minutes, and the scale bars represent 10 µm. The videos each start immediately after laser ablation (0.0 min).

## Source data

Source Data Fig. 1 All numerical data and statistical outputs related to Fig. 1.

Source Data Fig. 2 All numerical data and statistical outputs related to Fig. 2.

Source Data Fig. 3 All numerical data and statistical outputs related to Fig. 3.

Source Data Fig. 4 All numerical data and statistical outputs related to Fig. 4.

Source Data Fig. 5 All numerical data and statistical outputs related to Fig. 5.

Source Data Fig. 6 All numerical data and statistical outputs related to Fig. 6.

Source Extended Data Fig. 1 All numerical data and statistical outputs related to Extended data Fig. 1.

Source Extended Data Fig. 2 All numerical data and statistical outputs related to Extended data Fig. 2.

Source Extended Data Fig. 3 All numerical data and statistical outputs related to Extended data Fig. 3.

Source Extended Data Fig. 4 All numerical data and statistical outputs related to Extended data Fig. 4.

Source Extended Data Fig. 5 All numerical data and statistical outputs related to Extended data Fig. 5.

Source Extended Data Fig. 6 All numerical data and statistical outputs related to Extended data Fig. 6.

## Data Availability

All numerical source data are available in the supplementary data file. All other data supporting the findings of this study are available from the corresponding author on reasonable request. Source data are provided with this paper.
